# Anomalous Origin of the Right Coronary Artery from the Left Sinus of Valsalva: A Possible Trigger for Ventricular Arrhythmia

**DOI:** 10.3390/diagnostics16131986

**Published:** 2026-06-26

**Authors:** Małgorzata Zalewska-Adamiec, Michał Łuczaj, Jakub Bondaruk, Kacper Falkowski, Emil Julian Dąbrowski, Marcin Kożuch, Sławomir Dobrzycki

**Affiliations:** 1 Department of Invasive Cardiology, Internal Medicine with CICU and Laboratory of Hemodynamics, Medical University of Bialystok, 15-089 Bialystok, Poland; e.j.dabrowski@gmail.com (E.J.D.); marcin.kozuch@umb.edu.pl (M.K.);; 2 Department of Invasive Cardiology, Internal Medicine with CICU and Laboratory of Hemodynamics, University Hospital in Bialystok, 15-089 Bialystok, Poland; michalluczaj098@gmail.com; 3 Faculty of Medicine, Medical University of Bialystok, 15-089 Bialystok, Poland; 43267@student.umb.edu.pl (J.B.); 43805@student.umb.edu.pl (K.F.)

**Keywords:** coronary anomalies, AAOCA, AAORCA, coronary angiography, CCTA

## Abstract

Coronary anomalies in the form of anomalous aortic origin of the coronary arteries (AAOCA) are rare, but they may cause sudden cardiac death during physical activity in individuals under 35 years of age. We present the case of a 51-year-old man diagnosed with a coronary artery anomaly—anomalous origin of the right coronary artery (RCA) from the left coronary sinus (AAORCA). The patient complained of palpitations during exercise, and ventricular arrhythmia was detected during an exercise electrocardiographic stress test. During coronary angiography, selective cannulation of the RCA was unsuccessful despite the use of multiple catheters. Therefore, coronary computed tomography angiography (CCTA) was performed, which revealed a high-risk anatomical variant of AAORCA.

**Figure 1:**
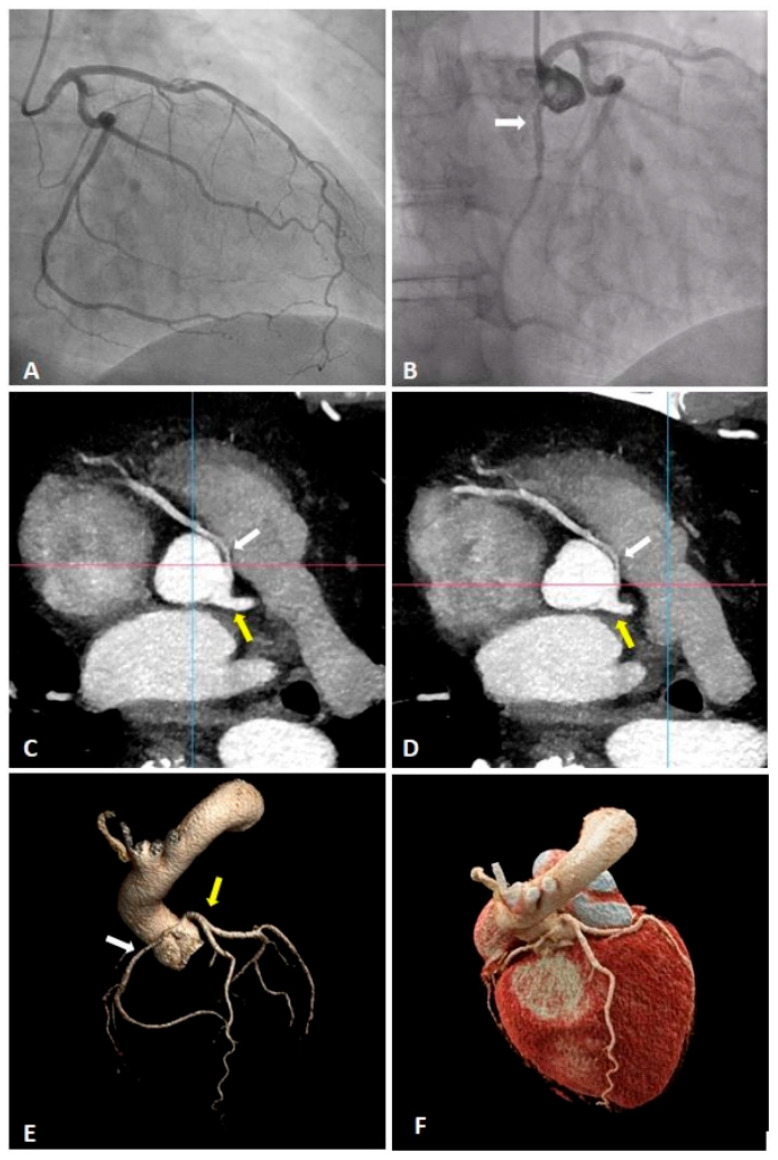
Anomalous aortic origin of the coronary arteries (AAOCA) is a congenital coronary artery anomalies that consists of an abnormal origin of the coronary arteries from the aortic root. AAOCA is rare. Its estimated prevalence is <1% in the general population but is higher among patients with other congenital heart defects. The increasing use of advanced cardiac imaging techniques has led to more frequent detection of these anomalies. AAOCA is more commonly observed among young (<35 years) victims of sudden cardiac death, often during or shortly after exercise. Although many patients are asymptomatic, symptoms associated with AAOCA may resemble those of other cardiovascular diseases, making it difficult to make an accurate diagnosis [1,2,3]. A 51-year-old patient with heart palpitations during exertion and exertional dyspnea (NYHA class II) was admitted to the Department of Invasive Cardiology for further diagnostics. Additionally, the patient had COPD, and multiple cardiovascular risk factors were identified, including a long history of cigarette smoking, obesity, hypertension, type 2 diabetes mellitus, and hyperlipidemia. At admission, the patient was asymptomatic in good general condition. An ECG, 24 h ECG monitoring and echocardiography examination showed no abnormalities. Ejection fraction was 55%. The patient underwent an exercise stress test according to the Bruce protocol. During the second stage of the test, isolated premature ventricular contractions were initially observed, followed by episodes of ventricular bigeminy and an increasing number of ventricular couplets. Therefore, the test was terminated at 7 METs (normal: 10 METs). During the exercise test, the patient reached 84% of the predicted maximum heart rate, with only a slight increase in blood pressure to 129/65 mmHg. The patient did not report anginal chest pain, and no significant ST-segment depression was observed on the ECG recording. Due to the presence of ventricular arrhythmias during physical exercise, the patient underwent coronary angiography, which revealed non-significant changes in left coronary artery (LCA) and anomalous origin of the right coronary artery arising from the left Valsalva sinus (**A**,**B**, white arrow). Due to an unsuccessful attempt at selective cannulation using multiple catheters and inadequate visualization of the right coronary artery (RCA) during coronary angiography, coronary computed tomography angiography (CCTA) was performed. CCTA revealed an anomalous origin of the right coronary artery (AAORCA) from the left aortic sinus, directly anterior to the origin of the left main coronary artery. The proximal segment of the RCA shows mild narrowing at its origin as it courses between the aorta and the pulmonary conus at the level of the pulmonary valve. The proximal segment has a smaller diameter, averaging 1.6 mm, compared with approximately 2.4 mm in the distal RCA (luminal caliber reduction <50%). Otherwise, the RCA is patent and shows no stenosis (**C**–**F**, RCA—white arrow, LCA—yellow arrow). Based on the CCTA findings indicating a high-risk anatomical variant, the Heart Team qualified the patient for surgical treatment. The patient declined the proposed cardiac surgical procedure and remains under follow-up at the Cardiac Surgery Outpatient Clinic. An AAORCA from the left coronary sinus is considered the most common coronary anomaly with a prevalence of 0.23–0.3% and is regarded as a high-risk anatomy in cases of interarterial course [1]. The preferred non-invasive imaging modality for visualizing the anatomy of the proximal segments of the coronary arteries and assessing key high-risk factors is CCTA [2]. According to the 2020 ESC guidelines, surgical treatment is recommended in symptomatic patients with typical anginal chest pain accompanied by objectively documented stress-induced myocardial ischemia or the presence of high-risk anatomical features [3]. The patient was found to have a high-risk coronary anomaly that did not present with typical anginal chest pain, but rather with a less typical presentation of exercise-induced ventricular arrhythmia. Given the arrhythmia observed during the exercise stress test, we concluded that the palpitations reported by the patient during physical exertion may have been episodes of ventricular arrhythmia, including ventricular tachycardia. The symptoms were most likely caused by the interarterial course of RCA and the resulting transient myocardial ischemia occurring during physical activity, which constituted an indication for surgical treatment. AAOCA presents in many variants, resulting in variable clinical courses and treatment approaches and current guidelines are not specific, causing each case to be treated individually.

## Data Availability

Original data supporting the reported results are available by contacting the authors.
